# Acute Fibrinous and Organizing Pneumonia Associated With Allogenic Hematopoietic Stem Cell Transplant Successfully Treated With Corticosteroids: A Two-Patient Case Series

**DOI:** 10.1177/2324709616643990

**Published:** 2016-04-13

**Authors:** Lam-Phuong Nguyen, Stella Ahdoot, Narin Sriratanaviriyakul, Yanhong Zhang, Nicholas Stollenwerk, Michael Schivo, Richart Harper

**Affiliations:** 1 University of California, Davis, CA, USA; 2 VA Northern California Health Care System, Mather, CA, USA

**Keywords:** AFOP, ARDS, allogenic stem cell transplant, BOOP, diffuse alveolar damage

## Abstract

Acute fibrinous and organizing pneumonia (AFOP) is an extremely rare, relatively new, and distinct histological pattern of acute lung injury characterized predominately by the presence of intra-alveolar fibrin and associated organizing pneumonia. AFOP may be idiopathic or associated with a wide spectrum of clinical conditions. It has a variable clinical presentation from mild respiratory symptoms to that similar to the acute respiratory distress syndrome. Currently there is no consensus on treatment, and corticosteroids previously were of unclear benefit. To date, there are less than 40 cases of AFOP reported in the literature and only one has been linked to hematopoietic stem cell transplantation. Here we report the first case series of 2 patients who developed AFOP following allogenic stem cell transplant that were successfully treated with high-dose corticosteroids.

## Introduction

Acute fibrinous and organizing pneumonia (AFOP) is an extremely rare diffuse parenchymal lung disease that was first described by Beasley et al^1^ as a possible variant of diffuse alveolar damage (DAD), bronchiolitis obliterans with organizing pneumonia (BOOP), or eosinophilic pneumonia (EP). It is now understood to be a distinct form of lung injury that is characterized pathologically by the presence of intra-alveolar fibrin and organizing pneumonia in a patchy distribution. AFOP has been observed in patients with collagen vascular disease, various infections, occupational exposures, or adverse drug reactions,^1,2^ making it difficult to identify clinical characteristics that are specific to AFOP. Here we report the first case series of 2 patients who developed AFOP following allogenic hematopoietic stem cell transplant (HSCT), which suggests AFOP may be more common in this patient group than previously recognized.

## Case Report

### Patient 1

**Figure 1.**
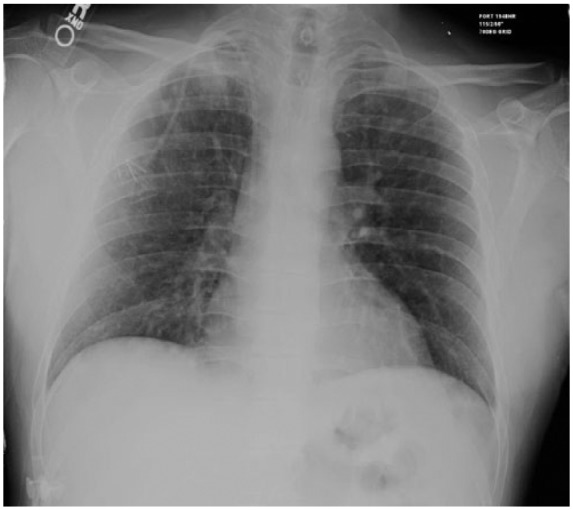
Admission imaging. Chest X-ray with evidence of bilateral airspace opacities.

**Figure 2.**
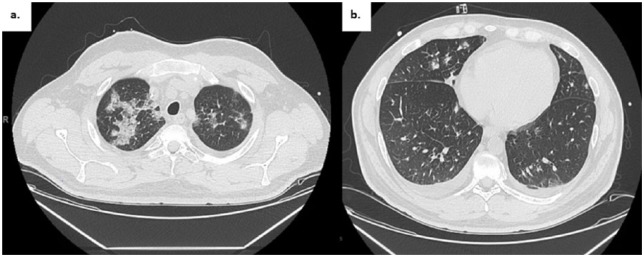
Admission chest CT. (a) Apical 1.25 mm cut showing widespread multifocal patchy opacities, most prominent at the apices. (b) Bibasilar with scattered opacities.

**Figure 3.**
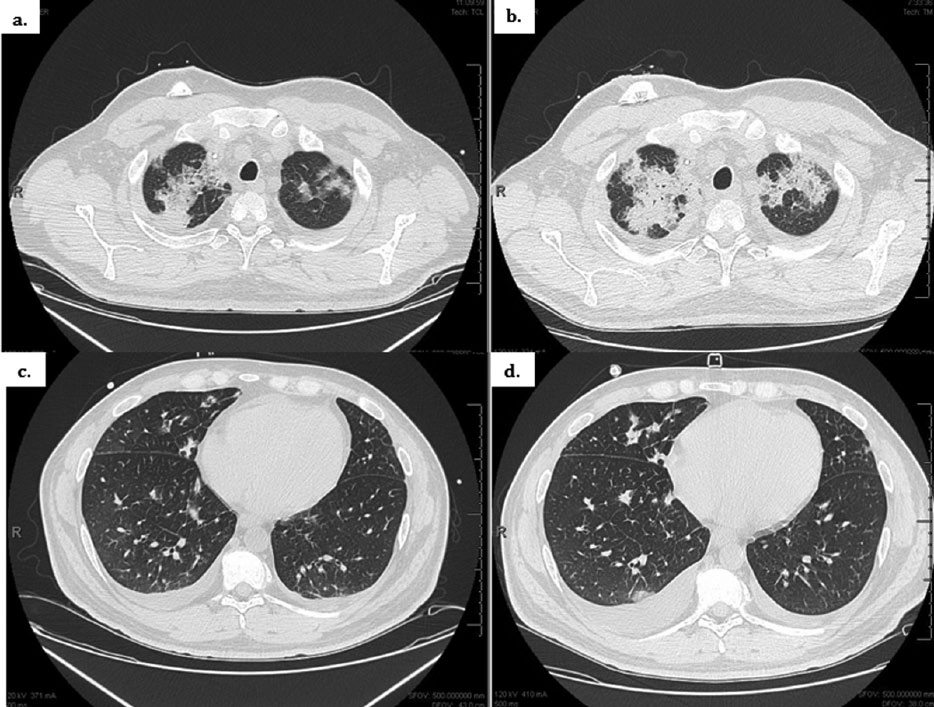
Comparative chest CTs. Admission CT scan (left; [a] upper lung zone, [c] lower lung zones). CT scan 9 days later (right; [b] upper lung zone, [d] lower lung zone) with worsening bilateral patchy and confluent airspace opacities.

A 39-year-old Caucasian man with acute B-cell lymphoblastic leukemia presented 6 weeks after allogenic HSCT. He presented with 1 week of cough and fevers with stable vital signs and an oxygen saturation of 96% on room air. Pulmonary exam was notable for faint crackles at the right apex. Laboratory studies revealed a normal white blood cell (WBC) count and differential. Chest X-ray and chest computed tomography (CT) scan demonstrated bilateral airspace opacities (Figures 1-3).

Bronchoscopy with bronchoalveolar lavage (BAL) of the right upper lobe and transbronchial biopsy of the right lower lobe were performed on hospital day 2. BAL cell count showed 605 WBC/mL with 76% histiocytes, 19% polymorphonuclear leukocytes, and 6% lymphocytes. Bacterial, fungal, viral, and acid fast bacilli studies from BAL were all negative. Transbronchial biopsy was nondiagnostic. All other extensive infectious workup was unremarkable.

**Figure 4.**
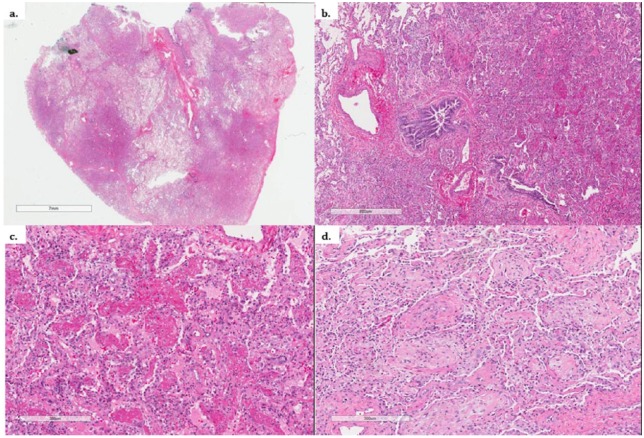
Histopathology of Patient 1. (a) Low magnification showing patchy, nodular intraluminal involvement. (b) Prominent fibrinous exudate in peribronchiolar parenchyma. (c) Higher magnification showing the fibrinous exudate within alveolar spaces. (d) Focally associated with organization.

Despite administration of broad-spectrum antibiotics and antifungals, the patient had worsening cough, dyspnea, and progressive hypoxemia, and therefore underwent surgical lung biopsy on hospital day 9 with pathology consistent with AFOP (Figure 4).

### Patient 2

**Figure 5.**
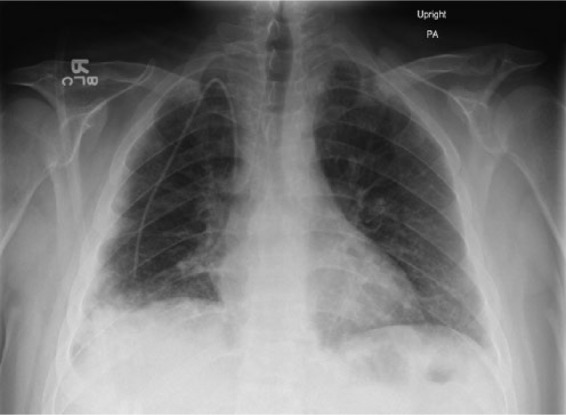
Admission chest X-ray of Patient 2. Bilateral nodular densities, most prominent in the periphery.

**Figure 6.**
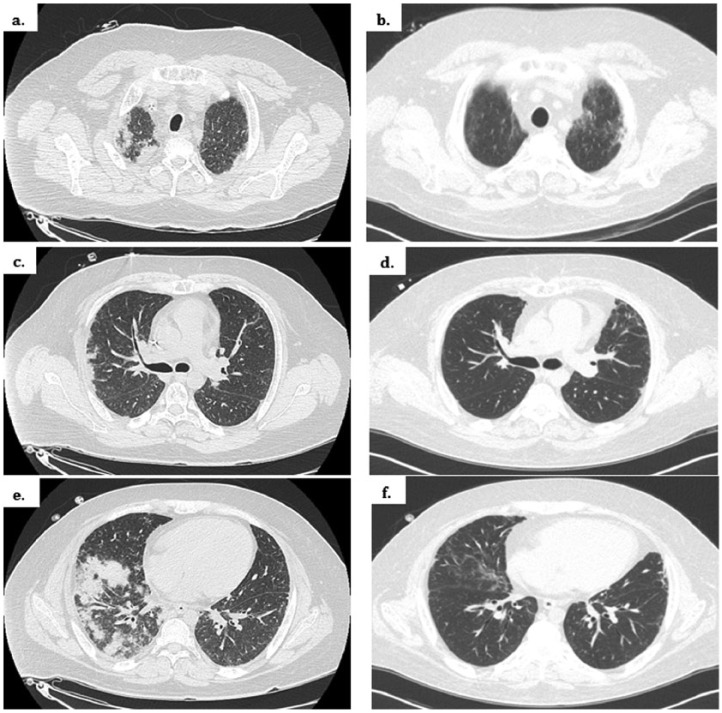
Radiographic comparison before and after corticosteroid therapy. Admission CT scan (left; [a] upper lung zone, [c] middle lung zone, [e] lower lung zone). Two months after high-dose corticosteroid therapy with marked improvement (right; [b] upper lung zone, [d] middle lung zone, [f] lower lung zones).

A 25-year-old Hispanic man presented 25 days after allogenic HSCT for relapsed B-cell lymphoma with persistent fevers of 102°F, progressive dyspnea, and cough. His chest imaging demonstrated multifocal pulmonary nodules, as seen in Figures 5 and 6.

**Figure 7.**
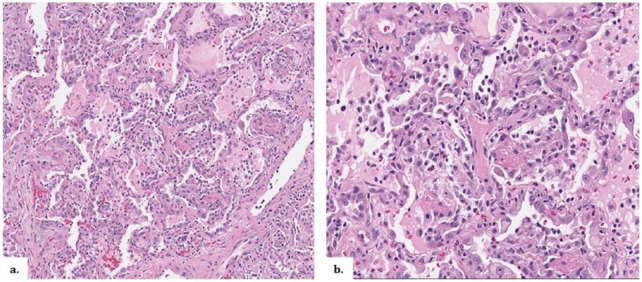
Histopathology of Patient 2. Biopsy results of right upper, middle, and lower lobes reveal diffuse acute fibrinous and organizing pneumonia (a) as well as an acute and organizing stage of diffuse alveolar damage (b).

He underwent bronchoscopy with BAL of the left lower lobe; WBC count was 970/mL (4% polymorphonuclear cells, 41% lymphocytes, 38% histiocytes, and 17% macrophages). Bacterial, fungal, viral, and acid-fast bacilli cultures from BAL were all negative. The patient underwent a surgical biopsy on hospital day 21. Pathologic examination revealed diffuse AFOP as well as an acute and organizing stage of diffuse alveolar damage (Figure 7). Following surgical lung biopsy, the patient developed the acute respiratory distress syndrome (ARDS) requiring ventilatory support.

Both patients were started on corticosteroids at 1 mg/kg of prednisone equivalence with clinical and radiographic response (Figure 6). After more than 6 months, both patients had full resolution of their pulmonary symptoms and were maintained on minimal prednisone doses (<5 mg daily).

## Discussion

AFOP is an extremely rare pathologic entity with unclear etiology, vague clinical presentation, and no consensus treatment with potential deadly consequence. This spectrum of disease currently does not fit within the definition of ideopathic interstital pneumonias based on the American Thoracic Society and European guidelines.^3^ To make it more confusing, most reported cases of AFOP have no identifiable risk factors, and the diagnosis of AFOP is similar to other forms of acute lung injury, often mimicking developing ARDS. Of the 17 cases of AFOP reviewed by Beasley et al, 6 cases had no identifiable association while the other 11 cases were believed to be associated with collagen vascular disease (3), amiodarone (1), *Haemophilus influenza* (1), *Acinetobacter* sp (1), lymphoma (1), and occupational exposures (including hairspay, coal mining, construction work, and zoological work^1^). Subsequent case reports since Beasley et al publication have linked AFOP to H1N1 infection, undiffentiated connective tissue diseases, bone marrow transplant, and others.^4,5,6,7^ Symptoms are nonspecific and include fever, cough, and dyspnea. Radiographic findings include bilateral diffuse migratory alveolar opacities distributed peripherally.^1,2,7,8^ There are 2 reported patterns of disease progression: a fulminant illness with overlapping features of ARDS which carries a much higher mortality and a more subacute illness with spontaneous recovery.^1,9^

Histologically, the AFOP pattern can be difficult to distinguish from other acute lung injury patterns of DAD, OP, or EP, especially on smaller biopsy specimens. Intra-alveolar fibrin, a dominant finding in the AFOP pattern, can also be seen in DAD, OP, and EP. AFOP pattern differs from DAD, OP, and EP by the presence of prominent organizing intra-alveolar fibrin, the lack of hyaline membrane, and rare numbers of eosinophils. A surgical biopsy is recommended for a diagnosis of the AFOP pattern. The key features of AFOP, DAD, COP, and EP patterns are summarized in Table 1.^1,10^

**Table 1.** Histologic Features of AFOP and Other Acute Lung Injury Patterns.

Predominant Histologic Distinction	AFOP	DAD	OP	EP
Intra-alveolar fibrin deposition	Yes, prominent	+/−, focal	+/−, focal	Yes, prominent
Intra-alveolar fibroblastic plugs (Masson bodies)	No	No	Yes, prominent	+/−, focal
Hyaline membranes	No	Yes, extensive	No	+/−, focal
Eosinophils parenchymal infiltrates	Rare	Rare	Rare	Yes, extensive

Abbreviations: AFOP, acute fibrinous and organizing pneumonia; DAD, diffuse alveolar damage; OP, organizing pneumonia; EP, eosinophilic pneumonia.

Currently, there are no established guidelines for the treatment of AFOP. Compared to OP, AFOP responds less reliably to steroids and portends a higher mortality rate. Among the 17 patients in the Beasley et al^1^ study, 7 patients received corticosteroids but only one survived. Other immune-modulators, such as cyclophosphamide, azathioprine, and mycophenolate mofetil, have been used in combination with corticosteroids in individual case reports with varying degrees of success.^11^ ARDS and respiratory failure requiring mechanical ventilation was primarily a predictor of poor outcome in the majority of case reports.^1,6^ In contrast to the other reported cases of AFOP following HSCT who progressed and died, our patients had excellent clinical responses to corticosteroids. Both patients presented with a subacute to acute illness, with patient 2 progressing to respiratory failure and ARDS requiring mechanical ventilatory support. However, despite having clinical features of ARDS, patient 2 was quickly liberated from mechanical ventilation. The use of corticosteroids in ARDS has been controversial and potentially harmful when given late in the course (>14 days), though our patient with AFOP had an excellent clinical response to high-dose steroids. Whether the underlying pathophysiology of AFOP makes patients more responsive to corticosteroids versus patients with infection-associated ARDS is unclear.

We believe that early diagnosis with prompt initiation of corticosteroid therapy may explain the excellent clinical responses in these AFOP cases. Also, early surgical lung biopsy should be considered in ARDS cases following HSCT who develop unexplained pulmonary infiltrates with poor response to empiric antimicrobial therapy. A biopsy to determine if they have AFOP, a condition likely amenable to steroid therapy, may change the course of treatment.

## Conclusion

AFOP is a rare, relatively new, and distinct histological pattern of acute lung injury with variable presentation and a high mortality. Early recognition and prompt diagnosis is essential, including consideration of surgical lung biopsy. Treatment with steroids should be considered, despite patients presenting with ARDS, if the underlying histological pattern is that of AFOP. To our knowledge, we report the first case series of AFOP presenting with hypoxic respiratory insuffiency progressing to ARDS following HSCT successfully treated with steroids.
